# Dr. Henry Neville

**Published:** 1918-09

**Authors:** 


**Dr. Henry Neville** died at his home in Jamestown July 16, age 78. He graduated from the Homeopathic Hospital College of Cleveland in 1872.

